# White Adultocene. Rethinking modernity through figures of the Child in the history of racial oppression

**DOI:** 10.3389/fsoc.2025.1482987

**Published:** 2025-07-02

**Authors:** Claudia Mock

**Affiliations:** ^1^Interdisciplinary Centre for European Studies (ICES), Europa University Flensburg, Flensburg, Germany; ^2^Collaborative Research Centre “Re-Figuration of Spaces” (CRC 1265), Technical University of Berlin, Berlin, Germany

**Keywords:** anti-black racism, childhood, adultism, whiteness, colonialism, Anthropocene, modernity, sociology

## Abstract

This paper explores how the figure of the Child has been used to uphold colonial anti-Black racial oppression. By examining adultism—the subordination of Children within the Child-adult binary—I trace its roots to Western philosophical ideas about nature. I furthermore show, how these ideas of nature informed racism within the modern constitution, where Children and Black people have been framed as “incomplete” or “not fully human”, revealing important intersections between racial and age-based inequalities. I introduce the concept of *white adultism*—the racialized separation of “being human” from “becoming human”—as a key feature of modernity and the Anthropocene. Recognizing this challenges the universalizing language used in the social sciences when discussing the “human” as the dominant force in this geo-social epoch. To critically engage with the colonial legacies within Western theories of modernization and to advance discussions on adultism in decolonial studies, I propose the notion of *becoming(s) in figuration*, which moves beyond fixed and developmental imaginaries of “being” to rethink the entanglements of race and age in the Anthropocene.

“Children […] are in most absolute subjection to him or her, that so bringeth them up, or preserveth them. And they may alienate them, that is, assign his or her dominion, by selling, or giving them, in adoption or servitude to others; or may pawn them for hostages, kill them for rebellion, or sacrifice them for peace, by the law of nature, when he or she, in his or her conscience, think it to be necessary.”

— Hobbes (2004 [1640]) (written in 1640, The Elements of Law Capt. 23, Section 8)

“Tribes in their infancy are to be brought to self-awareness through the demanding influence of the spiritually matured European in word and deed.”

— B. Herold (1894) (Translated from 1894, Die Behandlung der Afrikanischen N^****^, p. 11)

## 1 Introduction

The quotations from Hobbes and Herold indicate the historical role of adultism, so adults' supremacy over whomever is not considered adult, in the social construction of both, Children^1^ and Black people^2^. For sociological theory they point to the intersectional categories of age and race as historically entangled forms of othering and social differentiation. For the experience of Children and Black people this form of *social* differentiation goes beyond the *social* itself, as they both belong or are made belong to social groups, which were/are rendered outside of the realm of being fully human. Throughout the European enlightenment era and its modern constitution both, Children and Black people where scientifically conceptualized within a in a state of becoming—a naturalization which up until today strongly conditions their societal position. Therefore, historically adultism, is more than the relics of Thomas Hobbes “Elements of Law”, which up until today counts as one of the most influential theories of the social contract. It also manifested beyond the family, in ideologies of race and practices of racial oppression. It can be described as a general order of differentiation in colonization, where Black people were held inferior to White Europeans. Hence adultism must be *firstly*, reflected in its entanglements with whiteness as a collaborator in the colonization of both mind and matter, which *secondly* finds both its sources and resources in the modern philosophical constitution and as such—so as a white form of governing difference (e.g., Child/adult, Black/white)—has to be *thirdly* highlighted as an integral part of anthropocentrism.

As I will in the following elaborate, this white power of governing differences between, both nature and culture and being and becoming is up until today reproduced by the sociological tradition that still relies on the conceptions of human nature derived from the sciences, such as modern political philosophy (for example Thomas Hobbes, John Locke, Jean Jacques Rousseau, Étienne Bonnot de Condillac and Julien Offray de La Mettrie) and also propelled the growing dichotomization of nature and culture and human and non-human and its exploitation in the practices of “colonial earth-writing” (Yusoff, 2018, p. 2). Therefore, to decolonize sociological thought, it is necessary to also dismantle the historical relations between the dualisms they created, and which were used as forms of othering. One example of such othering that is central to this paper is the cruel act of declaring Black people as others—uncultured, uncivilized and therefore inhuman—which allowed the West to trade them as commodities during the transatlantic slave trade.

In considering the historical interplay of whiteness, adultism, modernity and anthropocentrism my primary objective is to emphasize the necessity of engaging with age as a marginalized category of social inequality within the matrix of domination (Collins, 1990) to comprehend historical processes of colonial anti-Black oppression and their theoretical continuation in the sociological tradition. The figure of the child and age as a category of social inequality proposed here as a fruitful category for decolonizing sociology. I argue that adultism also informs the postcolonial criticism of modernization theories, which are, as Bhambra (2007, p. 2) writes, characterized by “ideas of rupture and difference”. I see these characteristics both, challenged but also continued in the current Anthropocene debate: Challenged by its focus on nature-cultures and the more-than-human, which hardly allow for earth writing that relies upon rupture and difference. Continued by the attempts of its own periodisation, which reproduces the ideas of rupture and difference inherent in modernization theories, of which some take their starting point with the Industrial Revolution in the late 18th and early 19th centuries, triggered by the invention of the steam engine around 1784 (see Crutzen and Steffen, 2003, p. 61). By conceptualizing historical processes based on such ruptures and differences (that relate to Europe as the birthplace of modernity), not only the Eurocentric and white gaze is reproduced, but also the adultist logic of differentiating between being and becoming is reinforced. This is because, as Bhambra puts it with reference to Wallerstein for modernization theories, they are all characterized by “historiography, the parochiality of its universalism, its assumptions about (Western) civilization, its Orientalism, and its attempts to impose a theory of progress (Wallerstein, 1979, p. 94)” (Bhambra, 2007, p. 4).

In that context I see White adultism, as an integral aspect of anthropocentrism. Of white anthropocentrism to be precise and which I will elaborate in Section 3.1 (Who is the Anthropos?). Framed as “White [A]dultocene”, a term I borrow from Rollo (2016, p. 242), I do not seek to provide new concepts of anthropocentrism, but to deconstruct the common definitions through the lenses of Whiteness and adultism. The conjunction of the concepts of Whiteness, Adultism and Anthropocentrism serves me here to challenge the “continued privileging of the West as the ‘make' of universal history” (Bhambra, 2007, p. 2) because it points to both, the general eurocentrism of such a historical imagination together with its parochiality (Whiteness and Eurocentrism) and the specific mode of operating difference as a means of oppression enacted by constructing superiority via the category of age as a marker for developmental stages and generational orders, but also for the historiography rooted in the eurocentrism of modernization theories—understood as transformation and progress (adultism). Such notions of transformation, as I will elaborate in Section 3.2, follow a linear and developmental understanding of historical progress that remains with difference and rupture—a social construction of universal time, which also adultism relies on. Understood as such, White adultism is central to the modern constitution—a claim that I will subsequently fill with historical examples, when looking at the figures of the Child in the history of colonial, anti-Black racial oppression. As such it is central for the project of decolonizing the sociological tradition and hinder its reification of the discourse of the “West and the Rest” (Hall, 1994, p. 137) which functions as a representing system which was established in the course of European enlightenment, which claimed that the European was the most “most advanced type of society” and “Europeans were the pinnacle of human achievement” (Translated from Hall, 1994, p. 140). This is because the social sciences up until today heavily rely on the heritage of modern philosophy, which facilitated and perpetuated not only the governance of differentiation between nature and culture or being and becoming, but also broadly served to legitimize the differentiations between human and non-human, constructing binaries between humans themselves, e.g., Child vs. adult, white vs. Black and their connotation of underdeveloped vs. developed or uncivilized vs. cultured.

## 2 Being vs. becoming: 'wild' differentiations

The literature on colonial subjectivity and oppression frequently references the various portrayals of the colonized subject as a Child by white supremacist ideology (Fanon, 2008 [1961]; Hall, 1994; Hartman, 1997; Wynter, 2003). These examples illustrate how the trope of the Child was used to subordinate people racialized as Black, who were denied the status of full human beings, when compared to be at the same developmental stage with Children. Based on this infantilizing form of othering, they were treated and traded as commodities during the slave trade. In this context, the figures of the “feral,” the “savage,” and the “wild,” also associated with the Child, have historically been used to naturalize and legitimize the subordination and suppression of Black people. The construction of the Child as a creature of nature, still free of culture and the connection of that nature to ideas of the feral, savage, and wild, was strongly influenced by philosophical contributions of the modern era in Europe that encompass larger metaphysical considerations about the nature of being vs. becoming. The image of the Child as feral, savage and wild was largely shaped by European philosophy and appeared in works such as “Locke's tabula rasa, Condillac's gradually animated statue, […] LaMettrie's ‘homme machine”' (Yousef, 2001, p. 247), Hobbes Childhood, as a period of servitude and Rousseau's (1761 [1755]) Origins of Inequality Among Men. Even though the concept of nature differed among these thinkers, all of them “relied heavily on the Child as representing a nature that needed to be developed to become truly human” (Kromidas, 2014, p. 427). This philosophy of nature provided adults with the authority to protect, control, discipline, and speak on behalf of children and up until today constitute the Child/adult binary in which children are in a state of becoming, while adults count as accomplished beings.

The concepts of nature that were proposed by these thinkers also circulated during the period of European expansion and were employed to position racialized others as inferior to white Europeans legitimizing their project of civilization. As Purtschert (2012, p. 861) argues for Rousseau and Hobbes, “their conceptions of the state of nature are constitutively linked to imaginations of the savage that emanate from contemporary colonial discourses”. During the same period and culminating in the 19th century, that is viewed as the era that invented Childhood (Ariès, 2007 [1987]), the legitimacy of scientific racism, which posits that corporeal markers such as skin color or other perceived aspects of non-white or non-European appearance are racially significant, was growing. As such Childhood became a “site in which the ontological certainty of race as natural type is sustained” (Kromidas, 2014, p. 423). As Ashcroft posits, it is not mere coincidence that the Child/adult binary, and the Child itself as the (physical) Other and in need of protection and education, emerged concurrently with scientific racism. As such it forged a morality that legitimized the act of suppression as a form of protection.

“As a child, the colonial subject is both inherently evil and potentially good, thus submerging the moral conflict of colonial occupation and locating in the child of empire a naturalization of the ‘parent's' own contradictory impulses for exploitation and nurture. […] This ability to absorb contradiction gives the binary parent/child an inordinately hegemonic potency.” (Ashcroft, 2001, p. 36, 37)

Ashcroft's research on colonialism and missionarism as collaborative projects of mental control in the form of discipline and pedagogy reconstructs links between the historical power of the imperial and pedagogical discourses of representing the Other, which, “included racist imaginations of what being a full human (which is to assume of course, a white adult) means.” (Biswas, 2022, p. 314). In this context, Albert Schweitzer, a famous figure of colonialism, who was a German doctor and researcher who founded a hospital in the Central African country of Gabon, also performs and legitimizes racial oppression by constituting a similar ontological asymmetry between Europeans and Africans through the figure of the “older brother”.

“The N^****^ is a child. Without authority, nothing can be done with a child. So, I have to create the socializing formula that expresses my natural authority. To the N^******^ I have coined the word for this: I am your brother, but your older brother” (translated from Schweitzer, 2005, p. 325 ff).

Biswas (2022, p. 341) aptly frames this type of subordination as the “misopedy of philosophical racism”. Within this misopedy, rationality—which stood as the human marker by which (white) humans distinguished themselves from other living beings—was not accounted for “[n]on-European ‘others' [who] were consequently branded as sub-humans.” (ebd., p. 314). Biswas further notes that Western culture also categorizes itself in this way when she points to the “adultist logic” (Biswas, 2022, p. 342) within the mechanisms of Western (colonial) power through the governance of difference that lies in the entangled dichotomies of child vs. adult, nature vs. culture, and becoming vs. being, and notes that “[w]hile such imaginations are problematically applied to non-Western cultures, it is firstly problematic that such imaginations have been applied to children and childhood in e.g., Europe itself (!)” (Biswas, 2022, p. 342).

### 2.1 Intersections of race and age

What is clear from the illustrations above is that both Childhood and Blackness are sites of “naturalized violence and servitude” (Rollo, 2018, p. 307), and people positioned within these sites are and have been depicted as being in a current or permanent state of becoming, outside the realm of being fully human. Although, this points to a somewhat common fate of Children and people racialized as Black, their experience of subordination and oppression cannot be compared. Therefore, the basis on which difference is discussed here is theoretical rather than material. It is important to note that in the context of slavery, the experience of subordination and oppression was accompanied by the exploitation of labor and the extraction of natural resources, and thus appears as very different from the forms of oppression and subordination that adultism causes for the experience of Childhood it is also important to keep in mind that there is a certain anthropological difference that plays into this intersectional relationship: according to Gehlen (1940), humans are born as so-called “deficient beings” and are therefore dependent on the affection and care of adults. This means that they also benefit more from the protective function of the social category of Child, which is not the case for the racialized, infantilized adult. In this context, Rollo, with reference to Frederick Douglas, also draws attention to an essential and complex difference that separates not only the fate assigned by the category of Child between minors and racialized adults, but also between white and Black Children. In the former constellation, the Child is assigned to the status of becoming only for a certain biographical period, while for the latter, in the colonial logic of differentiation, “there would be no emancipation from the color of childhood [...]” (Rollo, 2018, p. 313).

Nevertheless, we need to admit, that we know little about the historical situation of children, as they are missing from the field of oral histories and primarily have been kept silent in the historical discourse. Their invisibility also lies in the fact that most marginalized groups become visible because they speak up for themselves first, which in the case of children does not lead them to be heard, but to be seen as not yet able to stand in for their rights. While many Black and decolonial writers critically point to the mechanisms of oppression through infantilization in colonizer/colonized relations, the Child-adult binary remains predominantly naturalized. In this context, Kromidas (2014, p. 426) notes somewhat cynically that “[a]t one time or another, so-called savages, racialized Others, women, children and animals have all been conscripted outside the boundaries of the human'. Only animals and children remain”. Along with Kromidas, there is a small but growing number of scholars who emphasize the need to liberate Children from their othered position as well (Nandy, 1984; Ashcroft, 2001; Kromidas, 2014; Rollo, 2018; Biswas, 2022).

By looking the historical entanglements of the social categories of age and race I want to point to adultism, which must be accounted as a “basic form of oppression […] for it teaches everyone how to be an oppressor and makes them focus on the exercise of raw power rather than on volitional humanness” (Pierce and Allen, 1975, p. 266), and that as such it has historically served not only to construct the Child/adult binary, but also served to constitute Otherness connected to race. The hierarchical thinking that results from this form of othering is also evident in the sociological tradition, for example in the context of Orientalism. Stuart Hall in that context mentions the developmental logic that even lies at the base of the works of capitalism-critical thinkers such as Marx and Engels, who denied that, e.g. Asian or Islamic societies would be able to develop the same progressive and dynamic capitalist structures, which they saw as “necessary conditions for the transition to [Europe's] capitalism and modernity” (translated from Hall, 1994, p. 116).

## 3 We have never been white and adult

After providing insights into the figure of the child as it has been instrumentalized within the history of colonial anti-Black oppression and deriving the historical relation of the intersectional categories of age and race from these insights. So, after highlighting the interplay of adultism and Whiteness, in the following I will elaborate on their entanglements with modernity and anthropocentrism. I will start with the notion of so-called “developmental countries”, as it demonstrates how relevant the mechanisms of power through the governance of difference between being and becoming are until today. They show that the idea of development, deeply rooted in modern philosophy and white European historicism is still an integral part of the global economy that perpetuates social inequalities to this day. The notion of developing countries is a product of colonial earth-writing, which, in the logic of capital accumulation, orders and thus hierarchizes the world as evolving from “primitive” and “simple” to “advanced” and “complex”—a line of differentiation that parallels the dichotomies of being and becoming, as well as nature and culture. The prevalence of such concepts continues to rely on historicism and its Eurocentric and linear understanding of social change. As such, it constructs the Global South as deficient and backward and is additionally (re)produced within the methodologies of social science and humanities research, for example in the form of “the provincialism of European universalisms” (Patel, 2014, p. 609) and through the blindness to “epistemic inequalities” (Amelina et al., 2021, p. 305, 310 with reference to Spivak, 1988).

To address problems like these, Latour (1993) initiates in “We Have Never Been Modern” a rethinking of the modern constitution inherited from the seventeenth century and shaped by figures such as Boyle and Hobbes. He criticizes their “purifying discourse” (Latour, 1993, 42; 139) of a dualistic representation of nature and culture and sees “the very notion of culture [as] an artifact created by bracketing Nature off” (Latour, 1993, p. 104, capitalization as in the original). Latour argues that despite ongoing co-production (hybridity of nature and culture), modernity remains blind to this interaction. Latour suggests that while we've never been modern, we've adopted the perception that we are. From the perspective of adultism, we could likewise argue that we've never been adult, we've just adopted the perception that we are. And this perception is not at all innocent, because “[o]ne society—and it is always the Western one—defines the general framework of Nature with respect to which the others are situated” (Latour, 1993, p. 105), thereby (re)producing certain aspects of racism that continue to link the figure of the Child and its canonical representations of savagery, wilderness, and wildness to Black communities and spaces. This makes it furthermore necessary to note that also Whiteness plays a role in the same narrative, as we've never been white, we have just adopted the perception that we are. Thus, the modern constitution, and with it the history of colonial earth-writing, can be deconstructed as one that regulates the difference between becoming and being.

Furthermore, if we sharpen the lens that Latour offers here toward the lengthy philosophical discourse on the “nature of man”, it appears that the Child/adult binary is closely tied to the nature-culture divide. As such, however, it is missing from discussions on anthropocentrism, even though they rely heavily on Latour's hybridity approach to nature and culture (Latour, 2005, 2013), which aims to establish a “symmetrical” science (Latour, 1993, p. 103ff)—one that overcomes its own construction of a dichotomy between nature and culture. In doing so, the discussions also somewhat reify the parallel dichotomizing connection that links culture to humans and nature to non-humans. But, as the above illustrated insights into the governance of difference of being and becoming employed in adultism and acted out in racism have shown, the line of differentiation also crosses humanity itself. While this crossing of lines is already drawn on deriving of the grammar of geology, which Yusoff (2018) described as a basis for exploitive and extractive economies of subjectivities under colonialism, the same crossing of lines based at the foundation of sociology itself, remains barely visible. Therefore, to redeem such a symmetrical sociology, we have to start by overcoming the dichotomy of being and becoming. While the interest in Children and Childhood within the Anthropocene debate is generally a growing (for a more detailed literature review, see Sjögren, 2023), very few scholars set Childhood as an analytical category to explore the power structures within the processes that constitute the Anthropocene (Ashton, 2023), while so far none of them center the problem of adultism. The aim of this article is to contribute to this perspective by reflecting on the historical relevance of the Child-adult binary and adultism as a “basic form of oppression” that, like “sexism and racism” (Pierce and Allen, 1975, p. 266), historically conditioned anthropocentrism and continues to strongly shape unequal social positions, increasingly on a global scale.

### 3.1 Who is the Anthropos in a white Adultocene?

For rethinking modernity through figures of the child in their entanglement with the history of racial oppression, I am viewing the Anthropocene through the lens of adultism. This is what I frame with the notion of the “[A]dultocene”, which I borrow from Rollo (2016, p. 242). While Rollo used the term to point to the inadequacy of the term Anthropocene, which grammar suggests that all humans are in the same way part of the planet's exploitation—an assumption that he denies for children, I add a critical whiteness stance here to point to the same inadequacy that likewise counts for the people united and divided by the history of anti-Black racial oppression. By focussing on the impact of adultism within historical contexts of racial oppression this paper aims to contribute to the postcolonial strand of rethinking modernity, by tracing back historical moments in which racial othering meant dehumanizing—a process of oppression, so far little debated within discussions on Anthropocentrism.

By looking at adultism in its interplay with whiteness and modernity, I want to point out the European supremacy, which was enacted anti-black racial oppression, exploitation and extraction, and which was historically constitutive for the irreversible anthropogenic effects that the term Anthropocene alludes to. It should be made clear that my framing of the “white Adultocene” is not intended to replace current conceptions of the Anthropocene as they have been developed in various academic disciplines since its emergence in 2000 (Crutzen and Steffen, 2003). Rather, I would like to offer a lens through which we can critically engage with the production of social inequalities and to do so beyond the Eurocentric gaze. This use also makes it possible to emphasize other lines of social inequality that are historically co-produced by adultism, such as sexism: Marxist feminist scholars have also indirectly pointed to the ambiguous position of women within the dualism of becoming and being, criticizing the dualistic attribution of women to nature and men to culture (Ortner, 1972). Ambiguous because they are seen as somewhere between the status of becoming children and the being of men. Instead, women have the status of liminal beings. They mediate between the states of being and becoming. In Caliban and the Witch Federici's (2004) points out that this liminality ascribed to womanhood has been exploited based on the naturalized nature of women—precisely the womb, in which society is reproduced. In the European Enlightenment aera, the Child was accounted for as human capital—this ascribed a completely new role to both Children and women and forced women into reproduction (see Federici, 2004, p. 93ff.). This naturalization of the nature of women(hood) is still pervasive today and becomes visible in the overrepresentation of women in the sphere of reproduction, where their labor is unpaid (Meillassoux, 1972; Bock and Duden, 1977; Beneria, 1979; Dalla Costa and Dalla Costa, 1999; Katz, 2001). More broadly, these ways of governing difference, serve capitalism as a constitutive part of the Anthropocene era, which is fully dependent on the division of nature and culture and being and becoming and accordingly differentiates societies into racial, sexist and aged orders to organize labor processes for maximum capital acceleration.

“Europe's legacies of slavery, genocide and dispossession were facilitated by intellectual constructions of terra nullius, […] which in turn laid the groundwork for ongoing domination by nation states […]. Thus, at the heart of notions of modern democratic citizenship we find patterns of violent racialization, gender violence and ableism. Such is the world today, a world built entirely by adults for adults. The scale of our environmental impact has even begun to alter the Earth's natural processes, leading some to label our contemporary epoch the anthropocene. Yet it is more aptly called the *adultocene* since it is not the young who have wrought these drastic changes. The Earth is perfectly capable of recovering from the footsteps of children.” (Rollo, 2016, p. 242).

Rollo's positioning of Children in the Anthropocene can be transferred to the colonized Black, indigenous and People of Color. Ironically it is them missing from the human footstep that is currently debated within the Anthropocene discourse. So, who's footstep are we currently dealing with in the debates on anthropocentrism? During the time of slavery, racist industrial Europe could only flourish in the way it did because much of its acceleration was based on the exploitation of Black people's forced labor. Historically, capitalism relied on extremely large numbers of people who were denied full human status to legitimize their subordination and exploitation for the sake of growth. Therefore, it is necessary to clarify, who were these “humans or the anthropos [who] have become the strongest geological force (whose) earthly influence has set irreversible geochronological developments in motion, [to] the extent of which is unprecedented in the history of humankind and the consequences of which are still completely unforeseeable” (translated from Block, 2021, p. 203)? From an only natural science-based perspective the Anthropocene might indicate the period, in which humans have become the strongest geological force, which imposes irreparable anthropogenic effects on the planet, but from a sociological perspective, such a definition derives from the idea that all humans are equal.

## 4 Becoming(s) in figurations

We must, rather, abandon this apposition of Being and beings: renounce the fruitful maxim whereby Being is relation […].

  —Glissant (1997 [1990], p. 170)

Historical reflections about the meaning adultism within the history of racial oppression has been introduced here by means to challenge the idea, that historical processes derive from a point zero, just like the child that Locke conceptualized as a *tabula rasa* (as a blank slate), which still must become human (adult). Instead, to stay with Bhambra, I propose the potential to rethink modernity by reconceptualizing that very nature of the Child. Denaturalizing the Child will allow to further challenge white historicity, that sees the birth of modernity in the West and constitutes the rest of the world as in the aftershock of the rupture of its birth, which is believed to be left in difference. Denaturalizing the Child furthermore helps to counter modern ideas of parochiality and universalism, because it challenges the division of being and becoming for once and for all. Rethinking the nature of the Child in its binary to adults shakes up common concepts of subjectivation, which may team up with postcolonial concepts of historical process, such that of “‘connected histories' (Subrahmanyam, 1997)” proposed by Bhambra (2007, p. 11), “as a way of dealing with difference in the context of attempting to reconcile general categories and particular experiences” and “not to deny voice to those who were somehow ‘fixed' by physical, social and cultural coordinates” (Bhambra, 2007, p. 32). Through the lens of Childhood, these physical, social and cultural coordinates, look extremely fixed, by physical coordinates, such as the sphere of social reproduction,^3^ social coordinates, such as those deprived of rights and voice, and cultural coordinates, visible in the prevailing assumptions about what Children are or are not capable of at a certain age, which prescribes certain cultures to them and excludes them as possible participants and actors from others. As such these fixed figures of the Child demonstrate a suitable vantage point, from which fixed histories can be deconstructed to uncover their connectedness beyond white, adult and modern representations. What the figures of the Child in the context of adultism, as a form of othering and therefore fixing, offers to the post- and decolonial discourse that wish to rethink modernity is a chance for reconceptualizing the common logics at the basis of hegemonial concepts of subjectivation, which are underpinned by developmentalism. What I mean with this is that the post- and decolonial tools of rethinking modernity which theoretically work, to stay with Bhambra, on the level of “general categories”, could profit from tools that are precise enough to work on the theoretical level of “particular experiences” or maybe more general of existences. The tool that I want to offer here is a reconceptualization of subjectivation, away from developmental stages in which humans transform from lower to higher stages (Child/adult binary), toward a process of constant becoming in figurations—a notion which I relate to Haraway (2008, p. 244) understanding of worlding processes, which re-appear “[i]f we appreciate the foolishness of human exceptionalism then we know that becoming is always becoming *with*, in a contact zone where the outcome, where who is in the world, is at stake”.

### 4.1 Becoming(s)

To elaborate on this reconceptualization of subjectivation, I will start with “becoming(s)” and in the following give a brief insight in different but persisting conception of the Child as in a state of becoming with the aim to call for a positive reinterpretation of the “non-subject positionality of those excluded from full being” (Chipato and Chandler, 2022, p. 170). To make this comprehensible, it is helpful to look at the existing work in childhood studies, where the status of being vs. becoming is debated. Childhood research has traditionally relied on developmental psychology and age-based classifications, such as Freud's (1969 [1905]) Five Stages of Psychosexual Development and Piaget's (1936) Theory of Cognitive Development, which conceptualize Children as being in a state of becoming, generally fixed by their anthropological by their differences from adults. Since the 1980s, this conceptualization of Childhood has undergone a significant shift, partly initiated by the work of Ariès (2007 [1987]), which led to the view of Childhood as a social construct rather than a natural state, and initiated the recognition of Children's voice and agency, and thus the overcoming of their state(s) of becoming (Qvortrup, 1987; Bühler-Niederberger, 2005; Jenks, 2005; Zeiher, 1983; Hengst, 2013). In the 2000s, the New Wave of Childhood Studies emerged, criticizing the overemphasis on Children's voice and agency and arguing for a reconceptualization of Children's emotions and relationships, emphasizing their material and biological aspects and their connections to the non-human world (Horton and Kraftl, 2006; Kraftl et al., 2012; Kraftl, 2013; Spyrou et al., 2018).

These three strands of childhood research conceptualize Children and Childhood in very different ways, resulting in ambivalent views of Children's positions. While I favor the New Social Sciences of Childhood and the Sociology of Childhood for challenging the Child-adult binary, they still inadvertently reinforce it. By equating Children with adults, they empower Children, but they also perpetuate adult-centeredness and the idea that humanity and being is about completeness. In this regard, Kromidas (2014, p. 428) also notes that, “[d]espite the many laudable aspects of this deconstruction of the child, some of the core assumptions remain intact […] because their critique has not penetrated the deep humanist understandings of developmental theory”. Thus, instead of repeatedly debating the status of Children relative to that of adults, I propose to work the other way around, following Wall (2019, p. 4), who suggests that Childhoods become “prisms or microscopes through which to deconstruct historical expressions of adultism” by questioning the status of being associated with adulthood. What I am suggesting here is that we stay with the status of Children conceptualized as becoming(s) and instead ask if there is anyone or anything that is not becoming? When existence is framed as “becoming(s) in figurations” rather than “coming into being”, an alternative understanding of history as well as subjectivity is provided, that challenges ideologies of difference based on the idea of development and progress such as underpinning modernization theories. This alternative subjectivity can then be mobilized as a tool to get to these “particular experiences” or existences that Bhambra wishes to reconcile with the “general classifications” that together frame such connected histories. For sociology this concept also challenges the continuation of the relationship of its white modernist foundation and subjectivity. By proposing the idea of “becoming(s)” as a way of conceptualizing subjectivation processes, I intend no less than to replace the notion of “being(s)” in order to further substantiate alternatives to a sociality, which moves beyond the Child/adult—becoming/being binary and its developmental notion of existence. For this to happen, it seems important to continue the project of denaturalizing Childhood. One way to do this is to “refigure the status of the child through the tools of posthumanism” (Kromidas, 2014, p. 423) because, as Kromidas aptly puts it, it “serves to remind us that when the human is defined by an essence or quality rather than a shifting and ambiguous figure, it will invariably exclude some humans” (Kromidas, 2014, p. 426). The definition of the human as a “shifting and ambiguous figure” is what I want to redeem here with the notion of “becoming(s)”. I don't want to impose another ontology of being, but rather to add to the anti-ontological discourse of positionality (e.g. Barad, 2007) and, like Chipato and Chandler, 2022 with reference to Brown (2021), to point to the “immersion or submergence in being rather than one of cuts and distinctions which can only be part of discourses of control and domination” (Chipato and Chandler, 2022, p. 170).

### 4.2 Figurations

Temporal terms such as development, progress, and transformation are strongly linked to conceptualizations of modernity that imply that there are entities that are fixed in time, rather than understanding the entangled processes of complex relations—what I call figuration. The term figuration is borrowed from the processual sociology of Elias (1970, 1988) who uses it to explore the relational interactions of subject and society beyond an *a priori* subject-object dualism. Figurations thus describe processual and dynamic social relations that result from “interweaving contexts” (translated from Elias, 1970, p. 75) of different actors and as such connect to the symmetrical science advocated by Latour, as well as to calls to rethink the linear understanding of historical process based on rupture and difference underpinning modernity discourses. For Elias, these actors can be human and non-human. He thus rejects the separation of social and natural sciences, noting that it leads us to perceive the world as divided into “nature and society, nature and culture, object and subject, matter and mind” (translated from Elias, 1988, p. 58), rather than seeing their complex relations. Elias's concept of figuration therefore allows for an understanding of social processes beyond a linear understanding of time and beyond the limitation of only one possible narrative of history and can be linked to postcolonial concepts that try to grasp global historical processes, such as Bhambra's suggestion of connected histories. Figuration also serves as a methodological framework for grasping more than local processes because it offers “a conceptual answer to the [...] criticized static dichotomy of the local, the global, and the national” (translated from Löw et al., 2021, p. 11) and is therefore suitable to challenge parochiality. In contrast to the notion of development, which emphasizes progress, the notion of figuration is linked to process. As such, it can help us challenge the developmental logic of progress on which adultism as a marker of modernization is based. The concept of figuration gets by without having a point of departure and a point of arrival in the historical narrative. As such, this understanding of process can be used to work against social constructions of generational orders, and it also serves to overcome the dichotomy of being vs. becoming. From this point of view, being is part of all becoming and can no longer be understood as a fixed state of existence in which humans appear to be complete and superior to others. An understanding of history that works with the idea of process rather than progress beyond the dichotomy of nature and culture also counters the social constructions of difference and thus challenges racialization and racism as a way of othering through the governance of these differences. With the notion of “becoming(s) in figurations”, I would like to offer a concept that can be helpful to counter the adultist and white logic of development, identified here as a historical component of colonial anti-Black oppression as an integral part of Anthropocentrism. When both, historical as well as social change is understood as process, not progress, then being—understood as a static and fixed form existence is overcome, because everything and everyone is becoming. Becoming(s) in figurations then serves as a way of thinking historical processes further away from the logic of development that perpetuates patterns of racism in particular and conditions of social inequalities in general.

## 5 Sociological theory in the Anthropocene *quo vadis?*

This article has shown that white adultism and its ontological dualism between being and becoming plays a central role in the history of power through the governance of difference. Through the lens of Childhood, the article addressed some of the historical conditions of colonial racism and manifestations of whiteness, which are closely tied to social constructions of adulthood, some of which are rooted in modern European philosophy. By taking a closer look at the historical entanglements of social inequalities related to race and age, it has been possible to show how together they relate to the problem of adultism, which has emerged as a powerful means of oppression through the governance of difference. The historical entanglements of racism and adultism, illustrated here via age and race as intersected categories of social inequalities, have shed light onto a so far little recognized historical processes within the ongoing discursive reproduction of the ‘West and the Rest', which up until today lead to global inequalities, which are also racialized.

The notion of the “white Adultocene” shall capture this historical strand of governing difference through the division of becoming and being, which up until today lies largely for granted within the Child/adult binary, in which we know and reflect much about how young people are protected but keep silent about how they are at the same time oppressed. As I mentioned earlier, this oppression is impossible to compare with the lot of Black slaves, yet the logic of oppression in its binary of being and becoming continues, so that Children up until today hold utterly unequal positions to those who act in and rule and build capitalist societies, spaces, ecologies and relationships, such as those of the core-family. To critically reflect anthropocentrism through the lens of Whiteness and adultism also uncovers their entanglement with patriarchist structures. In this light the Anthropocene rather appears as a geo-social epoch of the European white male adult. This perspective highlights a so far little recognized intersectional matrix of anthropocentrism, that opens grounds for Black, post- and decolonial approaches to center race as a pressing category of social inequality in the Anthropocene debate. It offers a valuable opportunity to address racialization and racism, as well as other forms of othering such as sexism and ableism by dealing with its own problem of adultism. To provide theoretical substantiation for this, I proposed the notion of “becoming(s) in figurations”, which alludes to an alternative theoretical subjectivation framework as well as an alternative notion to social change and transformation, which together or separate can be applied to bridge Black, de- and postcolonial critique of the modern constitution to the Anthropocene debate and plead for a rethinking of the inclusiveness of its periodization's.

In light of these insights, I plead for the necessity to reconsider who is the Anthropos that we are currently speaking of, censure and condemn within the Anthropocene debate, which entail the latest discussions led at the UN Climate Change Conference in Baku in November 2024, where the discursive figure of the developmental countries is once again central. The discussion held there largely circled around the necessity to support places framed as such with funding from the industrialized countries to work against the social and environmental crises of the Anthropocene. The outcome of the conference, in which these industrialized countries pledged only $300 billion out of the $1.3 trillion needed, or < 2 per cent of their economic output, shows how these countries are still disregarded when it comes to global responsibility and perpetuating social inequalities. Last but not least, by drawing, perhaps somewhat, uncommon lines between the societal position of children and people racialized as others, I would like to both highlight the potential of the marginalized category of age for Black, critical race, as well as de- and postcolonial studies, and also place the figure of the Child more centrally in the Anthropocene debate and reflections of anthropocentrism linked to confrontations with colonial racialized subjectivity.^4^ Applied to sociological inquiry this means, that reflecting on the power of adultism, within the Anthropocene as a geo-social epoch and current alternative to grand narratives such as modernity or globalization also opens paths to build theoretical ground for more just, complex and inclusive historical narrations as well as the different forms of knowledge related to them.

## Footnotes

^1^“Child” and “Black,” as well as “Children” and “Black people” are different social constructs, which lead to different experiences for those who are positioned within one or even both constructs. To emphasize that they are moreover intersectional categories of social inequality (age and race) I capitalize both as well as the Other.

^2^The role of adultism in the constitution of race is not only limited to anti-Black racism. Yet, as the historical insights gathered here are based on Black perspectives my focus lies there. This focus is to be understood as a limit of my research and invites for perspective that seek to uncover the role of adultism in other contexts, in which the governance of difference is organized through a differentiation between being and becoming, such as in the case of sexism or Orientalism.

^3^This, of course, does not account for working children.

^4^I would like to express my appreciation to Janina Dobrusskin, Sophie Mélix, Jona Schwerer, Valentin Domann and Paul Welch Guerra, as well as to the reviewers for their feedback and critical discussion, from which the article has benefited greatly.
